# Is continuity of primary care declining in England? Practice-level longitudinal study from 2012 to 2017

**DOI:** 10.3399/BJGP.2020.0935

**Published:** 2021-05-05

**Authors:** Peter Tammes, Richard W Morris, Mairead Murphy, Chris Salisbury

**Affiliations:** Centre for Academic Primary Care, University of Bristol, Bristol.; Centre for Academic Primary Care, University of Bristol, Bristol.; Centre for Academic Primary Care, University of Bristol, Bristol.; Centre for Academic Primary Care, University of Bristol, Bristol.

**Keywords:** continuity of care, GP Patient Survey, longitudinal studies, multilevel model, preferred GP, primary care

## Abstract

**Background:**

Continuity of care is a core principle of primary care related to improved patient outcomes and reduced healthcare costs. Evidence suggests continuity of care in England is declining.

**Aim:**

To confirm reports of declining continuity of care, explore differences in decline according to practice characteristics, and examine associations between practice populations or appointment provision and changes in continuity of care.

**Design and setting:**

Longitudinal design on GP Patient Survey data reported annually in June or July from 2012 to 2017, whereby the unit of analysis was English general practices that existed in 2012.

**Method:**

Linear univariable and bivariable multilevel models were used to determine decline in average annual percentage of patients having a preferred GP and seeing this GP ‘usually’ according to practicelevel continuity of care, rural/urban location, and deprivation. Associations between percentage of patients having a preferred GP or seeing this GP usually and patients’ experiences with the appointment system and practice population characteristics were modelled.

**Results:**

In 2012, 56.7% of patients had a preferred GP, which had declined by 9.4 percentage points (pp) (95% CI = −9.6 to −9.2) by 2017. Of patients with a preferred GP, 66.4% saw that GP ‘usually’ in 2012; this had declined by 9.7 pp (95% CI = −10.0 to −9.4) by 2017. This decline was visible in all types of practices, irrespective of baseline continuity, rural/urban location, or level of deprivation. At practice level, an increase over time in the percentage of patients reporting good overall experience of making appointments was associated with an increase in both the percentage of patients having a preferred GP and those able to see that GP ‘usually’.

**Conclusion:**

Patients reported a steady decline in continuity of care over time, which should concern clinicians and policymakers. Ability of practices to offer patients a satisfactory appointment system could partly counteract this decline.

## INTRODUCTION

Patients in England are registered at one general practice at which they can consult with any doctor or GP. A core principle of primary care is continuity of care, which is usually defined as seeing the same doctor over time. Continuity of care is highly valued by patients and GPs,^1^ and linked with healthcare cost reduction^2^ and improved patient outcomes,^3^ such as patient satisfaction,^4^ reduced emergency hospital admissions,^5^^,^^6^ and reduced mortality.^7^^–^^9^ Although some patients might benefit from a ‘fresh pair of eyes’^10^ and seeing the same doctor increases waiting time,^3^ in general, these disadvantages of continuity of care are outweighed by the benefits mentioned above; as such, it is important to monitor continuity of care.

Although some studies suggest continuity of care in England is declining,^3^^,^^11^ few have explored the trend over recent years or whether the decline varies according to the characteristics of practices or their patient populations.^11^ Practice appointment systems could, perhaps, encourage patients to consult a specific GP or, conversely, prioritise access over continuity; in addition, practices with a high proportion of patients who have long-term health conditions or greater health needs than other patients are likely to have more patients with a strong preference for seeing a particular GP.^3^^,^^12^ The authors have previously shown that the NHS’s policy of allocating patients to a named GP did not appear to have the desired effect of preserving continuity of care.^13^

Examining patient and practice characteristics associated with aspects of relationship continuity, such as patients having a preferred GP and being able to see their preferred GP, could potentially further inform policy. The causes and consequences of a decline in the percentage of patients having a preferred GP might differ from those relating to a decline in the percentage of patients being able to consult their preferred GP. It is also important to note that a preferred GP (relationship continuity) does not necessarily equate with the GP a patient sees most often (longitudinal continuity), which has been the focus of much of the research on continuity that has been undertaken. This study aimed to improve the understanding of the time trend of relationship continuity of care, split into:

- having a preferred GP; and
- being able to see that GP.

The objectives were to:

1. Confirm reports of a decline in continuity of care;
2. Explore differences in decline, according to practice characteristics; and
3. Examine associations between changes in practice populations or appointment provision, and changes in continuity of care.

**Table:** How this fits in

Recent studies suggest continuity of care in England is declining and, as continuity of care is a core principle of primary care, this should concern clinicians and policymakers. Little is known about the trend in continuity of care over recent years. This study used aggregated practice-level data from repeated questions from GP Patient Surveys undertaken between 2012 and 2017, on having a preferred GP and seeing this GP ‘usually’; the data showed a decline over time for both indicators by approximately nine percentage points. This decline is visible in all types of practices, irrespective of baseline practice-level continuity, geographic characteristics, or level of deprivation. As practices with higher percentages of patients reporting a good overall experience of making appointments showed that more patients were ‘usually’ able to see their preferred GP, it appears that a satisfactory appointment system could help counteract a decline in continuity of care.

## METHOD

### Study design and setting

The GP Patient Survey (GPPS) is an independent annual survey of over a million patients across England, conducted by Ipsos MORI on behalf of NHS England. It aims to determine how patients feel about their general practice and its primary healthcare delivery. This study used a longitudinal design on GPPS data reported annually in June or July. The authors used data for each year from 2012 to 2017 whereby the unit of analysis was English general practices that existed in 2012.^14^ Practices were only included if their patients’ response rate was *≥*20% and none of the values on the characteristics included in the models used by the authors were missing. As a result, the number of practices included varied from a maximum of 7574 (91.7% of all operative practices in 2012) to a minimum of 6711 (90.2% of all operative practices in 2017). Weighted GPPS data were used; weights were generated to correct for the sampling design, reduce the impact of non-response bias, and to improve generalisability.^15^

### Outcome measures

Two questions from the GPPS that capture two different aspects of relationship continuity of care formed the basis of two separate outcome measures. The outcome measures were as follows:

- the percentage of patients answering ‘yes’ to the question about having a preferred GP, excluding those who responded that ‘there is usually only one GP in my GP surgery’ (Supplementary Table S1a); and
- the percentage of those patients with a preferred GP, who saw that GP ‘always’, ‘almost always’, or ‘a lot of the time’ (Supplementary Table S1b) — this is referred to as ‘usually’ seeing their preferred GP.

### Practice characteristics and differences in decline

The modifiers used in the models to explore differences in decline, according to practice characteristics, comprised practice-level continuity of care, location, and level of deprivation.

#### Practice-level continuity of care

This was used to explore whether declines in continuity varied according to whether a practice had, on average, a poorer or better continuity of care compared with other practices. Practices were categorised into quartiles according to the mean percentage of patients over the time period 2012 to 2017 who had a preferred GP. Similarly, this was also done for the mean percentage of patients who ‘usually’ saw their preferred GP. Quartile 1 represented the lowest percentage and quartile 4 the highest.

#### Location

Office for National Statistics data from 2011 were linked by general practice postcode to the GPPS to determine whether general practices were in rural areas, cities and towns, or urban conurbations.

#### Level of deprivation

This was measured using the Index of Multiple Deprivation score at practice level for 2012, provided by Public Health England.^16^ The scores were divided into quintiles, with quintile 1 representing the least deprivation.

### Measures of general practice population demographics and appointment provision

The following measures, calculated for each year, were used to examine associations between changes in practice patients’ characteristics or appointment provision, and changes in continuity of care:

- patients’ health status — the percentage of patients who indicated on the GPPS that they had a longstanding health condition;
- access to English general practice — the percentage of all GPPS responders who reported having a ‘very good’ or ‘fairly good’ overall experience of making an appointment; and
- sociodemographic characteristics — the percentage of patients reported to be aged ≥65 years, female, in full-time paid work or education, to have no religious affiliation, and identifying as African and Caribbean black, South Asian, or any other ethnicity that was not UK white (UK white ethnicity was the reference ethnicity in the analyses).

### Statistical methods

Multilevel analyses were conducted to:

- calculate change over time in the two continuity outcome measures;
- calculate differences between practices over time, by including an interaction between time and general practice characteristics; and
- examine the association of changes in practice population demographics with changes in continuity of care, whereby time (years) constituted the level-1 unit and general practices the level-2 units.

For all variables fitted in the last model above — with the exception of percentage of patients having a good overall experience of making appointments — the average value over the 6-year period was used, as it did not change substantially during that time (Supplementary Tables S1a and S1b). The percentage of patients having a ‘good’ overall experience of making an appointment did change, from 82.4% in 2012 to 76.0% in 2017. The authors conducted global tests to investigate whether decline in having a preferred GP and seeing a preferred GP usually differed between deprivation quintiles, practice-level of continuity of care quartiles, and locations.

**Figure 1.**
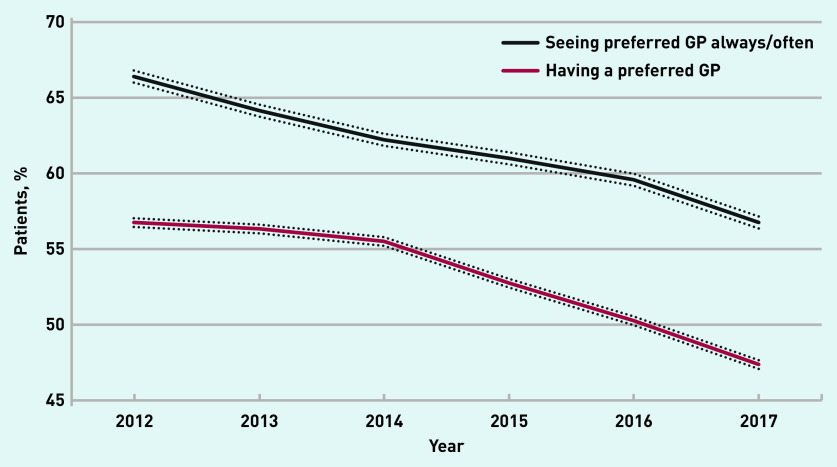
*Percentage of patients in English general practices having a preferred GP and seeing their preferred GP ‘always’, ‘almost always’, or ‘most of the time’, 2012–2017. Data calculated from GP Patient Survey responses. Dotted lines = 95% confidence intervals.*

**Figure 2.**
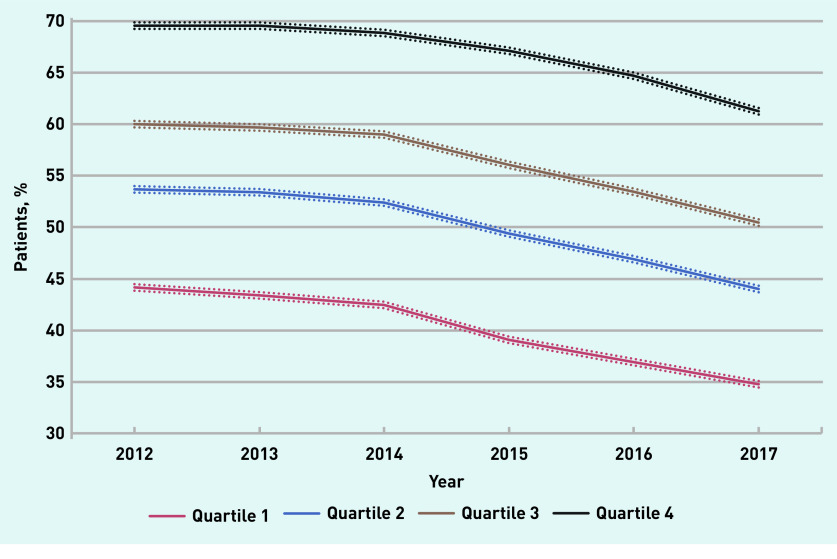
*Change in mean percentage of patients having a preferred GP over the years 2012–2017 (95% confidence interval [CI]), according to average percentage at the level of the practice during the period, divided by quartiles. Data calculated from GP Patient Survey responses. Dotted lines = 95% CIs.*

**Figure 3.**
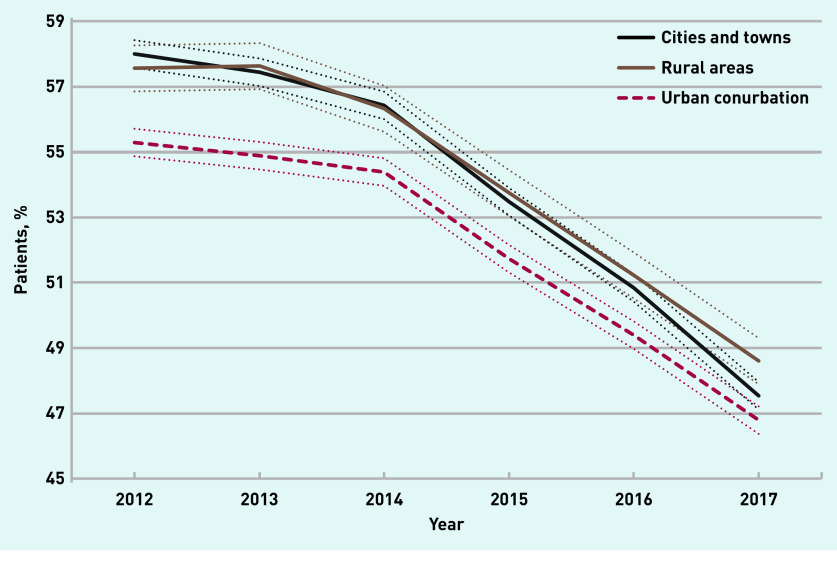
*Change in mean percentage (95% confidence interval [CI]) of patients in English general practices having a preferred GP by urban/rural location, 2012–2017. Data calculated from GP Patient Survey responses. Dotted lines = 95% CIs.*

**Figure 4.**
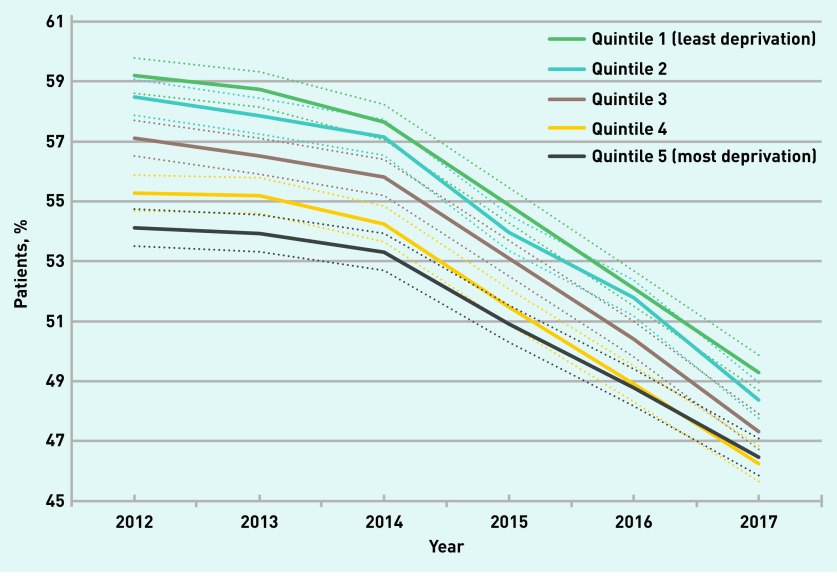
*Change in mean percentage (95% confidence interval [CI]) of patients in English general practices having a preferred GP by level of deprivation (quintiles), 2012–2017. Data calculated from GP Patient Survey responses. Dotted lines = 95% CIs.*

The third objective involved measuring the effect of these changes, at practice level, on having, or seeing, a preferred GP; therefore, as well as fitting the average over the 6 years for this variable (time average value), the yearly average per practice (that is, time-specific values) was also taken. This allowed the authors to model the effects of changes in overall experience of making an appointment, between and within practices, on changes in having, or seeing, a preferred GP. Adjusted regression coefficients, 95% confidence intervals (CIs), and *P*-values were tabulated for each predictor in this last model; the results of the first two models are presented graphically in Figures 1–4.

All analyses were undertaken using Stata/MP, version 16.1.

## RESULTS

### Confirmation of a decline in continuity of care over time

A multilevel analysis on having a preferred GP by survey year estimated that 56.7% of patients had a preferred GP in 2012. This declined by 9.4 percentage points (95% CI= −9.6 to −9.2) by 2017 (Figure 1). A similar analysis on seeing one’s preferred GP estimated that 66.4% of the patients with a preferred GP ‘usually’ saw this GP when consulting a doctor in 2012; by 2017, this had declined by 9.7 percentage points (95% CI = −10.0 to −9.4) (Figure 1).

### Exploration of differences in decline by practice characteristics

#### Having a preferred GP

The percentage of patients having a preferred GP declined in all four practice-level continuity-of-care quartiles; the decline in quartiles 1–3 was similar — around 9.5 percentage points — while the decline in quartile 4 was 8.3 percentage points (Figure 2).

In 2012, the percentage of patients having a preferred GP was lower in urban conurbations (55.2%) than in cities and towns (57.9%) or in rural areas (57.5%); these percentages declined by 8.5 percentage points, 10.5 percentage points, and 9.0 percentage points respectively (Figure 3). In 2012, the percentage of patients having a preferred GP was also lowest in those practices in areas of greatest deprivation (quintile 5, 54.1%) and highest in areas of least deprivation (quintile 1, 59.2%); these percentages declined by 7.7 percentage points and 9.9 percentage points respectively (Figure 4).

Global tests suggested these declines in having a preferred GP were statistically significantly (*P<*0.001) different among the practice-level continuity-of-care quartiles, practice locations, and deprivation quintiles, although these differences were modest in magnitude.

#### ‘Usually’ seeing preferred GP

A similar set of analyses on whether patients ‘usually’ saw their preferred GP also showed a decline among practices across all practice-level continuity-of-care quartiles (11.4, 11.5, 9.1, and 5.9 percentage points, respectively for quartiles 1, 2, 3, and 4), all locations (8.9 percentage points [rural areas], 11.1 percentage points [cities and towns], and 8.2 percentage points [urban conurbation]), and all levels of deprivation (9.5 percentage points for quintile 5 and 9.0 percentage points for quintile 1) (Supplementary Figures S1, S2, and S3).

Global tests suggested these declines were statistically significantly (*P<*0.003) different among the practice-level continuity-of-care quartiles, practice locations, and deprivation quintiles — although, again, these differences were modest in magnitude.

### Association of change in practice population characteristics or practice appointment provision with change in continuity of care

#### Having a preferred GP

Practices with higher percentages of patients with a preferred GP were associated with a higher percentage of patients who had a good overall experience of making an appointment (time average), were female, were aged *≥*65 years, and identified as South Asian.

Practices with lower percentages of patients with a preferred GP were associated with a higher percentage of patients who identified as UK white or as African/Caribbean black, were in full-time paid work or education, or who had no religious affiliation.

In practices in which the percentage of patients who had a good overall experience of making an appointment increased over the years (time specific), there was also an increase over the years in the percentage of patients who reported having a preferred GP (Table 1).

**Table 1.** Estimates of β-coefficients from multilevel regression models for the association between general practice characteristics and sociodemographic profile of patients and the percentage of patients in English general practices having a preferred GP, and the percentage of patients seeing their preferred GP ‘usually’,^a^ 2012–2017^b^

	**Patients had a preferred GP**						**Patients saw preferred GP ‘usually’^a^**					
	
	**Univariable**			**Multivariable**			**Univariable**			**Multivariable**		
			
	β**-coefficient**	**(95% CI)**	***P*-value**	β**-coefficient**	**(95% CI)**	***P*-value**	β**-coefficient**	**(95% CI)**	***P*-value**	β**-coefficient**	**(95% CI)**	***P*-value**
**Constant**				28.45	(20.96 to 35.93)	*P*<0.001				8.67	(0.52 to 16.82)	*P* = 0.037

**Year**												
2013 (ref 2012)	−0.39	(−0.61 to 57.00)	*P*<0.001	−0.18	(−0.39 to 0.04)	*P*= 0.109	−2.28	(−2.56 to −2.00)	*P*<0.001	−1.11	(−1.38 to −0.85)	*P*<0.001
2014 (ref 2012)	−1.22	(−1.43 to −1.01)	*P*<0.001	−0.86	(−1.08 to −0.65)	*P*<0.001	−4.20	(−4.49 to −3.93)	*P*<0.001	−2.26	(−2.53 to −1.99)	*P*<0.001
2015 (ref 2012)	−4.00	(−4.20 to −3.77)	*P*<0.001	−3.54	(−3.77 to −3.32)	*P*<0.001	−5.44	(−5.73 to −5.16)	*P*<0.001	−2.92	(−3.20 to −2.64)	*P*<0.001
2016 (ref 2012)	−6.48	(−6.69 to −6.27)	*P*<0.001	−6.02	(−6.24 to −5.80)	*P*<0.001	−6.86	(−7.15 to −6.58)	*P*<0.001	−4.35	(−4.61 to −4.07)	*P*<0.001
2017 (ref 2012)	−9.37	(−9.59 to −9.15)	*P*<0.001	−8.84	(−9.07 to −8.62)	*P*<0.001	−9.68	(−9.97 to −9.40)	*P*<0.001	−6.83	(−7.11 to −6.55)	*P*<0.001

**Practice–level variables**												
Cities and towns (ref urban conurbation)	1.96	(1.45 to 2.46)	*P*<0.001	1.31	(0.76 to 1.86)	*P*<0.001	2.58	(1.87 to 3.30)	*P*<0.001	−1.18	(−1.76 to −0.59)	*P*<0.001
Rural areas (ref urban conurbation)	1.97	(1.27 to 267)	*P*<0.001	−1.65	(−2.44 to −0.86)	*P*<0.001	9.55	(8.56 to 10.53)	*P*<0.001	−1.43	(−2.27 to −0.58)	*P*= 0.001
Low IMD score in 2012, quintile 2 (ref lowest IMD)	−0.72	(−1.45 to 0.07)	*P*= 0.052	−1.14	(−1.84 to −0.44)	*P*= 0.001	−1.32	(−2.36 to −0.28)	*P*<0.001	−0.30	(−1.05 to 0.44)	*P*= 0.420
Middle IMD score in 2012, quintile 3 (ref lowest IMD)	−1.92	(−2.64 to −1.19)	*P*<0.001	−1.72	(−2.48 to −0.95)	*P*<0.001	−4.00	(−5.04 to −2.95)	*P*= 0.013	0.11	(−0.70 to 0.92)	*P*= 0.787
High IMD score in 2012, quintile 4 (ref lowest IMD)	−3.39	(−4.12 to −2.66)	*P*<0.001	−2.63	(−3.51 to −1.76)	*P*<0.001	−6.17	(−7.22 to −5.12)	*P*<0.001	−0.15	(−1.09 to 0.78)	*P*= 0.748
Highest IMD score in 2012, quintile 5 (ref lowest IMD)	−4.16	(−4.90 to −3.41)	*P*<0.001	−2.96	(−4.05 to −1.86)	*P*<0.001	−8.01	(−9.08 to −6.93)	*P*<0.001	−0.03	(−1.21 to 1.15)	*P*= 0.955

**Patient–level variables^c^**												
Having good overall experience of making appointments (time–specific)	0.22	(0.21 to 0.23)	*P*<0.001	0.09	(0.07 to 0.10)	*P*<0.001	0.67	(0.66 to 0.68)	*P*<0.001	0.46	(0.45 to 0.47)	*P*<0.001
Having good overall experience of making appointments (time–average)	0.19	(0.17 to 0.21)	*P*<0.001	0.09	(0.06 to 0.11)	*P*<0.001	0.97	(0.95 to 0.99)	*P*<0.001	0.46	(0.43 to 0.49)	*P*<0.001
With longstanding health condition	0.24	(0.20 to 0.28)	*P*<0.001	0.07	(−0.00 to 0.14)	*P*= 0.051	0.32	(0.26 to 0.38)	*P*<0.001	−0.08	(−0.12 to −0.00)	*P*= 0.040
Female	0.22	(0.16 to 0.28)	*P*<0.001	0.15	(0.08 to 0.22)	*P*<0.001	0.23	(0.14 to 0.32)	*P*<0.001	−0.37	(−0.45 to −0.29)	*P*<0.001
Aged ≥65 years	0.37	(0.34 to 0.40)	*P*<0.001	0.41	(0.35 to 0.48)	*P*<0.001	0.62	(0.58 to 0.66)	*P*<0.001	0.30	(0.22 to 0.38)	*P*<0.001
African/Caribbean black ethnicity	−0.24	(−0.28 to −0.19)	*P*<0.001	0.02	(−0.05 to 0.08)	*P*= 0.621	−0.65	(−0.71 to −0.58)	*P*<0.001	−0.09	(−0.16 to −0.03)	*P*= 0.008
South Asian ethnicity	−0.01	(−0.03 to 0.02)	*P* = 0.608	0.16	(0.13 to 0.19)	*P*<0.001	−0.31	(−0.34 to −0.29)	*P*<0.001	0.04	(0.01 to 0.08)	*P*= 0.015
Non–UK white non–African/Caribbean black, non–South Asian ethnicity	−0.09	(−0.11 to −0.07)	*P*<0.001	0.11	(0.08 to 0.14)	*P*<0.001	−0.29	(−0.32 to −0.27)	*P*<0.001	−0.06	(−0.09 to −0.03)	*P*<0.001
In full–time paid work or education	−0.29	(−0.32 to −0.26)	*P*<0.001	−0.09	(−0.15 to −0.03)	*P*= 0.002	−0.30	(−0.34 to −0.25)	*P*<0.001	0.05	(−0.01 to 0.11)	*P*= 0.124
No religious affiliation	−0.09	(−0.12 to −0.07)	*P*<0.001	−0.08	(−0.12 to −0.04)	*P*<0.001	0.28	(0.23 to 0.31)	*P*<0.001	0.03	(−0.02 to 0.07)	*P*= 0.241

**Random components of variance**												
General practice level: intercept				87.60	(84.58 to 90.52)					93.34	(89.95 to 96.72)	
Year level: intercept				44.16	(43.51 to 44.80)					64.97	(64.01 to 65.94)	

**Statistics**												
*N*					44 002						41 962	
Deviance					3 107 97.18						3 105 95.57	
Intra–class correlation					0.66						0.59	

a *Collated responses: ‘always’, ‘almost always’, and ‘a lot of the time’.*

b *Based on responses from GP Patient Surveys undertaken from 2012 until 2017 inclusive.*

c *Percentage of patients. CI = confidence interval. IMD = Index of Multiple Deprivation.*

#### ‘Usually’ seeing preferred GP

A positive coefficient in Table 1 indicates a positive relationship, for example, practices with a higher percentage of patients having a preferred GP also had a higher percentage of patients having a good overall experience of making an appointment (time average coefficient 0.09 (95% CI = 0.07 to 0.10). Similarly, they also, for example, had a higher percentage of patients *≥*65 years. Practices with lower percentages of patients who ‘usually’ saw their preferred GP were associated with higher percentages of patients with longstanding health conditions and higher percentages of female patients. In practices in which the percentage of patients who had a good overall experience of making an appointment increased over the years (time specific), there was also an increase, over the years, in the percentage of patients who ‘usually’ saw their preferred GP (Table 1).

Regression coefficients for a good overall experience of making an appointment were substantially larger for ‘seeing’ a preferred GP than for ‘having’ a preferred GP, a difference not explained by the greater variation between practices for seeing a preferred GP (Table 1).

#### Post-estimation statistics

Residual plots were undertaken and showed that residuals for the final models were found to be normally distributed, although the small minority of practices with only one or two data points on the outcome measures had a slight tendency to show lower satisfaction than predicted (data not shown). Analyses were, therefore, repeated on those practices with in excess of two data points; the regression coefficients were, essentially, similar (data not shown).

## DISCUSSION

### Summary

The percentage of patients who had a preferred GP and were ‘usually’ able to see that GP declined substantially between 2012 and 2017. This decline was visible in all types of practices, irrespective of baseline practicelevel continuity, geographic characteristic, or level of deprivation. Although there was slight variation in the decline in continuity of care among practices according to practice-level continuity, location, and level of deprivation, the magnitude of the difference in the decline was small. An increase in the percentage of a practice’s patients that reported having good experiences of making appointments over time was associated, in particular, with an increase in the percentage who were able to see their preferred GP.

### Strengths and limitations

This longitudinal study used data spanning 6 years and included *>*90% of all operative English general practices; it involved repeated measures for patients having a preferred GP and patients seeing their preferred GP, together with a range of (potential) modifying and explanatory variables.

By excluding GPPS years for practices that had either a response rate of *<*20% or missing values for variables in the analyses, some practices with relatively more younger patients, more patients from an ethnic minority background, and located in more deprived areas were excluded (Supplementary Table S2). However, the analysis was repeated on practices with data for in excess of 2 of the 6 years; results relating to the study’s aims were, essentially, similar. Even after making exclusions, the available dataset was large, and the result of this was that many of the statistically significant associations reported were small.

There was a positive association between GPPS response rates and the percentage of patients having a preferred GP, which might reflect unmeasured confounding or selection bias. The GPPS questions were focused on a ‘particular doctor’ the patient usually prefers to see and how often a patient could see this doctor; the wording of these questions does not allow for an examination of more-complex aspects of continuity of care — for example, patients might prefer to see different doctors for the management of different conditions.^17^

Over the years, extended-hours consultations have been introduced, more GPs have been working part time, and GP workload has increased,^18^ all of which could affect continuity of care; however, it was not possible to include these issues in the models. Given the ecological nature of the data (aggregated to general-practice level), associations for individual patients cannot be inferred.

### Comparison with existing literature

Many studies on continuity of care have considered longitudinal continuity (seeing the same doctor), but it is arguable that it is more relevant to consider relationship continuity (seeing a preferred doctor). Levene *et al* combined the GPPS questions on having and seeing a preferred GP, and showed that the proportion of patients having a preferred GP and usually able to see that GP declined between 2012 and 2017; their study concluded that level of deprivation (in deciles) was not associated with a decline in relational continuity of care.^11^ The study presented here, however, investigated trends in having a preferred GP and usually seeing this preferred GP separately, and showed that both declined over time by approximately nine percentage points.

Forbes *et al* found that practices that had grown in population size between 2013 and 2018 had a greater percentage fall in continuity of care,^19^ supporting the conclusion presented here that the decline in continuity of care, which is visible in all types of practices, might vary according to practice characteristics.

Contrary to Levene *et al*, this current study found that the extent of decline differed statistically significantly between levels of deprivation (quintiles), although these differences were small.^11^

This study’s results concerning the patient characteristics associated with having a preferred GP — namely, being female, older, South Asian, not in full-time work or education, and having a long-term condition — were in accordance with those reported by Palmer *et al.*^3^ The authors of the study presented here also found an inverse relationship for patients who had no religious affiliation; which has not been reported by other studies.

In addition, the results concerning the patient characteristics associated with ‘usually’ seeing a preferred GP — being male, older, identifying as South Asian, or having long-term conditions — were also in accordance with those reported by Palmer *et al*.^3^ However, in the study presented here, no association with full-time work or education was found, but a negative association with being African/Caribbean black was identified.

### Implications for practice

A decline in continuity of care was identified in the first decade of this century after several major reforms had been introduced in UK primary care.^20^ The reported decline of *>*6 percentage points in patients being able to see their usual GP coincided with reforms that prioritised access to GPs over continuity of care. The introduction of the named GP scheme for older patients in 2014, and for everyone else the year after, did not improve continuity of care;^13^ assigning a named GP to a patient did not necessarily reflect which GP the patient had seen most often or take into account their preference for a certain GP. Current reforms include the introduction of primary care networks (PCNs), a core requirement of which is offering extended-hours access, shared across practices in a network — this policy may well further reduce continuity of care. A briefing from the Health Foundation on this stated that any evaluation strategy for the networks should include monitoring the effect on continuity.^21^

A rethink is needed regarding what is understood as continuity of care in a changing patient population and a rapidly evolving healthcare system. Accelerated by the COVID-19 pandemic, traditional face-to-face consultations are being replaced by telephone, video, and e-consultations.^22^^,^^23^ Patient access to care is increasingly triaged via algorithm-based or reception-led navigation, which directs patients to different health professionals, and advances in information technology are shifting some care to monitoring or information continuity through case managers and coaches/counsellors.^24^ This could result in reconsidering how to distinguish between different types of continuity of care — such as longitudinal, relational, informational, and managerial — and their mutual relationship,^25^^,^^26^ and the circumstances in which continuity of care most benefits patients’ health outcomes.

The identified association between satisfaction with making an appointment and the ability to see a preferred GP could suggest that improvements in the ability of practices to offer patients a good experience of making an appointment could partly counteract the decline in patients seeing their preferred GP; however, it is important to be aware that the reverse interpretation — in which people who were more likely to see their preferred GP were more likely to express improved satisfaction with the appointment system — is also possible. Patient satisfaction with the appointment system and patients’ ability to consult a preferred GP could reflect the organisation of the appointment system or pressures on the number of appointments available; this, in turn, may be related to practice workload and capacity.
